# Analysis of milk consumption and dairy products of the Russian population using an online survey

**DOI:** 10.1002/fsn3.3808

**Published:** 2023-12-18

**Authors:** Alla L’vovna Novokshanova, Natalia Olegovna Matveeva, Dmitry Borisovich Nikityuk

**Affiliations:** ^1^ Federal State Budgetary Scientific Institution ‘Federal Research Centre of Nutrition, Biotechnology and Food Safety’ Moscow Russian Federation; ^2^ Federal State Budgetary Educational Institution of Higher Education ‘The Vereshchagin Vologda State Dairy Farming Academy’ Vologda Russian Federation; ^3^ I.M. Sechenov First Moscow State Medical University of Ministry of Healthcare of the Russian Federation (Sechenov University) Moscow Russian Federation; ^4^ Peoples' Friendship University of Russia Named After Patrice Lumumba Moscow Russian Federation

**Keywords:** dairy industry, dairy products, milk, nutritional value, questionnaire

## Abstract

Over the past 20–30 years, the consumption of milk and dairy products in Russia has been steadily declining. An online survey was carried out to find out the reasons for this. The questionnaire was created on the Yandex forms platform with automatic counting of answers. The questionnaire contained 34 questions about the inclusion of dairy products in the diet, the reasons for refusing dairy products, the nutritional value of dairy products, etc. Statistical data processing was performed by the analysis of variance (ANOVA). The significance level was taken equal to 0.05.

## Graphical abstract

Over the past 20–30 years, the consumption of milk and dairy products in Russia has been steadily declining. An online survey was conducted to find out the reasons for this.

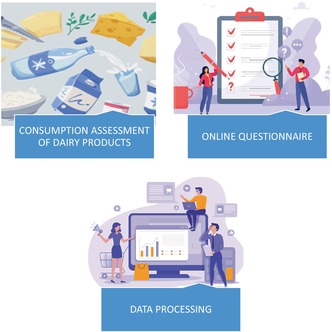

## 1 INTRODUCTION

The role of milk and dairy products in human nutrition is very high. In terms of nutritional value, milk serves as a source of essential nutrients for human development, since it contains easily digestible and balanced proteins, as well as fats, carbohydrates, and natural calcium. For this reason, dairy products are not only an obligatory part of the diet but are also used as a preventive and therapeutic agent for various diseases. T.K. Thorning and co‐authors (Thorning et al., 2016) concluded in their review that the consumption of milk and dairy products contributes to compliance with the recommendations on nutrients and can protect against the most common chronic diseases such as obesity, type 2 diabetes, cardiovascular diseases, colorectal cancer, bladder cancer, stomach cancer, and breast cancer. At the same time, as the authors note, the side effects of drinking milk and dairy products have been reported very rarely in studies.

The methodological complexity of organizing research on the impact of milk consumption on human growth and development lies in the fact that it is almost impossible to form control groups of subjects whose diets will lack dairy products for a long time.

Walter C. Willett and David S. Ludwig confirm the ambiguity of the conclusions about the role of dairy products in human health (Willett & Ludwig, 2020). In their opinion, the existing general recommendations to increase the consumption of dairy products to 3 or more servings per day are not fully justified, since the amount of milk that a person should consume will depend on individual circumstances.

Despite this, in most countries, there are formed ideas about the benefits of dairy products and preferences for their inclusion in the diet. S. Rosenberg and co‐authors (Rozenberg et al., 2016) presented a review containing information for health professionals to enable them to help their patients make informed decisions about consuming dairy products as part of a balanced diet. Similar medical and biological recommendations are valid in the Russian Federation (Recommendations on Rational Norms Order 2016).

In Russia, milk has traditionally been valued for its nutritional properties. In Ancient Russia, the main type of milk consumed was cow's milk. People used to say a cow in the yard, lunch on the table. The loss of a cow for a large peasant family was like a disaster.

The formation and development of the dairy industry in Russia began at the end of the 19th century. Until 1870, commercial dairy cattle breeding in Russia was concentrated in landowners' farms. The construction of railways in Russia in the second half of the 19th century improved conditions for the transportation of perishable dairy products to the markets of large cities—Moscow, St. Petersburg.

The dairy industry in Russia developed mainly in the direction of butter production. In 1901, Russia ranked second in the world in terms of butter exports. For many decades, butter in Russia has been one of the indicators of wealth and prosperity. This can be confirmed by the apt folklore expressions: “Cow's butter – eat for health”, “You can't spoil porridge with butter”, “To be rolling like cheese in butter,” and many others.

The issues of milk consumption and dairy products are of great importance not only for milk processing enterprises. For many countries, the dairy industry is a significant sector of the economy, creating jobs, and contributing significantly to the development of the state. In terms of nutritional value, milk and dairy products serve as a guarantor of essential nutrients for human development.

For these reasons, in most countries of the world, a lot of attention is paid to the dairy industry by the state. Тhe dynamics of the development of the industry, including the demand for dairy products by consumers, are monitored by the departments of agriculture, processing industry, and various analytical agencies.

For example, a UN study (FAO, Food and Agriculture Organization of the United Nations, 2014) revealed which countries are characterized by high levels of consumption of dairy products. In Finland, the Netherlands, Sweden, Montenegro, and Lithuania, an average person consumes more than 300 l of milk per year. At the same time, in countries such as Ireland and the Netherlands where milk consumption is traditionally high, the average life expectancy remains at 82 and 81 years, while in Russia it is only 72.4 years (PRODUKT.BY, 2019; Statistics and indicators. Regional and federal, 2020).

**FIGURE 1:**
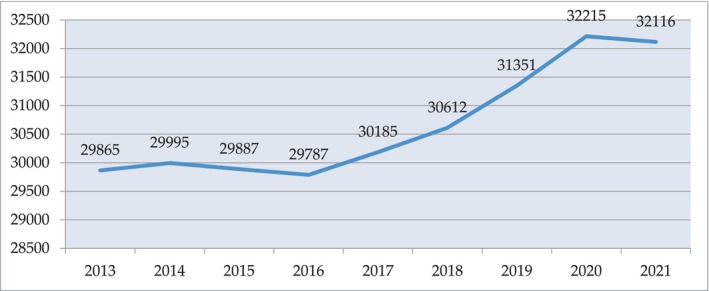
Milk production in the period 2013–2021 in farms of all categories in the Russian Federation, thousand tons.

In Russia, the milk processing industry experienced great difficulties in 1991–1996, when there was a sharp drop in gross milk production, which later was characterized by relative stability and averaged 30.7–30.8 million tons. Since 2017, there has been an increase in production of milk preparations (Figure 1).

Experts predicted that the growth of commercial milk production and the development of the dairy market in Russia in 2020 would continue (Dairy Intelligence Agency, 2020). The basis for this is the high level of investment activity in the industry and the continued support of the state. According to the PRODUKT.BY news portal, in 2018, state support for the dairy sector in Russia amounted to about 30 billion rubles (PRODUKT.BY, 2019). As a result, in 2020, the dairy industry of the Russian Federation showed record growth. The production of all key types of dairy products in 2020 increased to the highest levels in the history of modern Russia.

Despite this, our country has been ranked 35th in the world in terms of drinking milk consumption in recent years, 33rd and 18th in terms of cheese and butter consumption, respectively (ITR 45–2017, 2017). According to the daily specialized information and analytical agency, “Milknews”, the per capita consumption of milk and dairy products in the Russian Federation in 2021 amounted to 240 kg, which corresponds to 73.8% of the recommended norm (Milknews, 2021).

According to the data of the Center for the Study of the Dairy Market in Russia published in 2022, there is insufficient milk consumption in 46 regions of the country, which is home to more than 60% of Russia's population (Dairy Intelligence Agency, 2020).

The current situation worries not only dairy industry technologists due to a drop in demand for products and a slowdown in the development of the industry but also the medical community due to the deterioration of the nutritional value of the diets eaten by the Russian population (Novokshanova, 2019).

In an attempt to understand the current steady trend of insufficient dairy products in the diets of Russians, an online consumer survey was conducted.

## 2 MATERIALS AND METHODS

### 2.1 Experimental design

The purpose of the experiment is to identify the reasons for the decrease in consumption of dairy products among the population of Russia. The decline in demand for milk processing products made by industrial enterprises, which has been observed for 30 years, has a negative impact on the pace of development of the dairy industry.

The factors of biological variability in the composition of milk—as determined by the animal's breed, their stage of lactation, diet, welfare, and other factors—were not factored into our study and in the description of experimental results and conclusions. Despite the fact that each of the zootechnical factors can significantly affect the composition of milk, and this significantly distinguishes milk samples by individual animals from samples of precast milk, which is used for industrial processing at the plant, these indicators are averaged.

In Russia, when purchasing milk from producers, in accordance with the current documentation, organoleptic indicators (taste, smell, color, and consistency), protein content, fat, skimmed milk residue, and somatic cells are taken into account. Dairy plants are required to monitor physicochemical parameters (density, freezing point, titrated, and pH), and microbiological indicators (the number of mesophilic aerobic and facultative anaerobic microorganisms, *E*. *coli* group bacteria, pathogenic, including salmonella). In addition, the procurement provides for the determination of hygienic safety indicators in milk, which include heavy metals (Pb, As, Cd, Hg), pesticides, mycotoxins, dioxins, melamine, and antibiotics (levomycetin, tetracycline group, streptomycin, and penicillin) (CU TR 021/2011, 2011; CU TR 033/2013, 2013).

In the Russian Federation, the main volume received for industrial processing is cow's milk. Nevertheless, a number of plants make products from other types of milk: goat's, sheep's, mare's, camel's, buffalo's, or donkey's milk. In the case of processing these types of milk, appropriate identification indicators (organoleptic, physicochemical) and the same indicators of microbiological and hygienic safety as for cow milk are provided for them.

When purchasing milk, the acceptance of raw milk from individual producers is not excluded; the values of milk identification indicators obtained during individual milking may change in wide range if these indicators are not caused by animal diseases, which must be confirmed by acts (protocols) of control milking (CU TR 021/2011, 2011; CU TR 033/2013, 2013).

The method of online questionnaire was used in the work. The questionnaire was created on the Yandex Form platform, which allows for the automatic counting of responses. The survey was conducted between November and December 2020 and between February and March 2021. Participation was voluntary and individual. There were no restrictions on age, region of residence, level of education, income, or gender. A total of 220 people participated in the survey.

The questionnaire included 34 questions grouped into blocks. There were 12 questions in the questionnaire about the inclusion of dairy products in the diet, the reasons for refusing dairy products, the nutritional value of dairy products, and the preferred types and tastes of dairy products. Three questions were related to information about known cases of falsification of dairy products. Six questions were about the presence of lactose intolerance and allergies to milk proteins. Four questions were asked in order to find out from consumers the role of the price, the manufacturer's trademark, and the place of sale. The questionnaire also contained questions about the region of residence, the age of the respondents, and their employment. In some questions, multiple answers could be chosen.

When developing the questionnaire, we were guided by the experience and recommendations of experts in consumer surveys (Chon et al., 2021; Delorme et al., 2021; Dillman, 2000; Dolnicar & Grün, 2013; Keeter et al., 2015; Puleston, 2013). The questioning was preceded by an explanation of the purpose of the survey. The introduction contained the following text:

“Over the past 20–30 years, the consumption of milk and dairy products has been steadily declining in a number of countries. The dairy managers do not fully understand how this phenomenon can be explained, and so the following survey sets out to investigate the reasons for it.

Your opinion is highly valuable for us; thus, we would be grateful, if you could complete the questionnaire and share a link to this page via social networking websites.

The analysis of the survey findings will be published and open to the general public; please note that your responses will be kept anonymous. If you would like to receive the article, please leave your email address at the end of the questionnaire.

Thank you for your time and cooperation with us!”

The survey's completion time was not tedious and took no more than 10–15 min.

### 2.2 Statistics

Statistical data processing was carried out by the method of dispersion analysis (Analysis of Variation, ANOVA). When the null hypothesis was rejected, a multiple comparison of averages was performed using the Tukey test. The average values and standard errors were calculated using Microsoft Excel 2016 software. Excel programs were used to determine significant differences between the average values and plot diagrams, and obtain mathematical dependencies. The accepted confidence level did not exceed 5% (*p* < .05).

## 3 RESULTS

**FIGURE 2:**
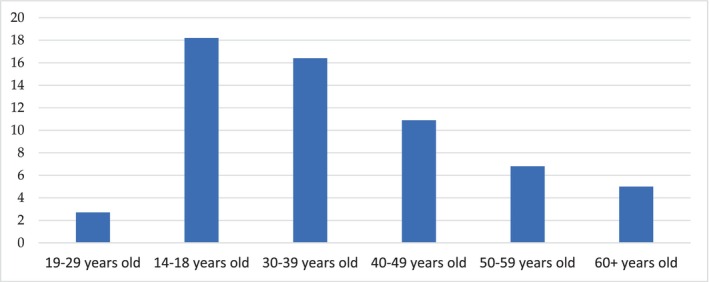
Age of the survey participants, % of the total number of respondents.

Participants were 160 females (72.8%, *p* < .001) and 60 males (21.8%, *p* < .001). A significant proportion of participants were in the age category up to 29 years (*p* < .001), but there was no significant difference in the number of respondents—adolescents aged 14–18 and adults aged 30–39 (*p* = .59). In addition, there was no significant difference among the number of survey participants aged 30–39 and aged 40–49 (*p* = .10), the number of survey participants aged 40–49 and aged 50–59 (*p* = .22), and the number of survey participants aged 50–59 and aged over 60 (*p* = .59). A general idea of the age of respondents is shown in Figure 2.

When determining the main employment of respondents, it was found that the main contribution to the frequency distribution of the occurrence of all respondents was made by students (42.0%) and working people (40.0%). Dispersion analysis revealed that the number of consumers of both these groups had significant (*p* < .001) differences from the other categories of respondents. Also, a reliable response (*p* < .001) was established for the group combining study and work (15.9%), and for citizens who neither study nor work (1.8%).

**FIGURE 3:**
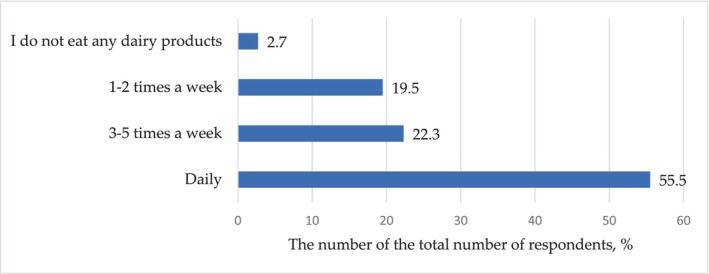
Distribution of responses (% of the total number of respondents) to the question: How often do you eat dairy products?

Of the entire audience, the overwhelming majority of consumers 96.4% include dairy products in their diet, which has a significant medicosocial and statistical response (*p* < .001). Regarding the question about the frequency of inclusion of dairy products in the diet, the answers were distributed, as shown in Figure 3.

According to these data, with a reliability (*p* < .001) much lower relative to the accepted level of significance, more than half of the respondents (55.5%) include dairy products in their diet daily. There is no significant difference between the answers to the question: “Do you include dairy products in your diet 1‐2 or 3‐5 times a week?” (*p* = .46). In total, 41.8% of consumers chose these two options. Consequently, dairy products are present in the diet of most respondents, several times a week.

**FIGURE 4:**
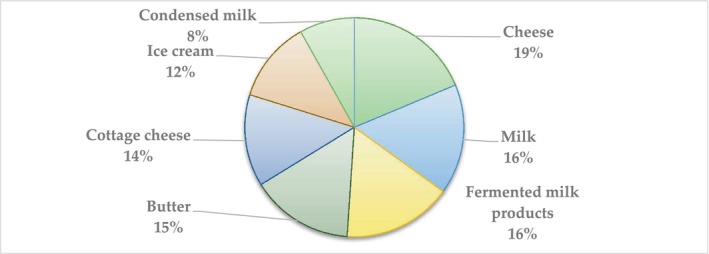
Distribution of responses (% of the total number of respondents) to the question: What dairy products do you include in your diet?

Regarding the question as to what dairy products participants include in their diet, the distribution of the occurrence of all responses is shown in Figure 4.

In terms of the frequency distribution, cheese, milk, and fermented milk products are in the greatest demand among consumers.

By analyzing the type of dairy products included in the diet, the null hypothesis can be rejected with respect to cheese and butter (*p* = .015), cheese and cottage cheese (*p* < .001), cheese and ice cream (*p* < .001), cheese and condensed milk (*p* < .001), as well as cheese and complete exclusion of dairy products from the diet (*p* < .001). In all these cases, consumers include cheese in their diet more often than other listed products.

However, there was no statistically significant response in the frequency of including cheese and milk (*p* = .10), cheese and sour milk products (*p* = .08), milk and sour milk products (*p* = .89) in the diet.

A significant statistical and medicosocial response (*p* < .001) was established between the frequency of inclusion of milk and ice cream in the diet, milk and condensed milk, fermented milk products and ice cream, fermented milk products and condensed milk, butter, and condensed milk, or complete exclusion of dairy products from the diet.

The consumption of ice cream and condensed milk (*p* = .007), as well as butter and ice cream (*p* = .035), had statistical significance.

There was no statistically significant response in the frequency of inclusion in the diet of milk and cottage cheese (*p* = .06), milk and butter (*p* = .42), butter and cottage cheese (*p* = .28), fermented milk and butter (*p* = .50), and fermented milk and cottage cheese (*p* = .08).

The next most popular product is butter. To a large extent, this can be explained by traditions.

**FIGURE 5:**
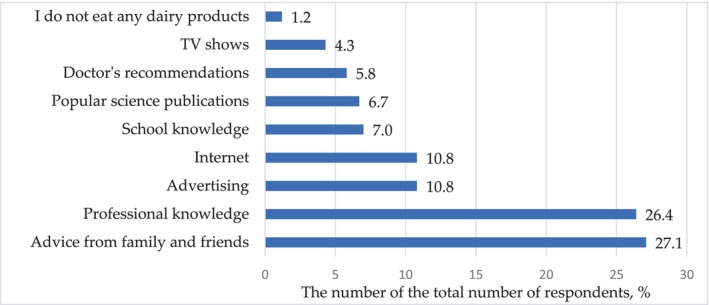
Distribution of responses (% of the total number of respondents) to the question: What kind of information do you consider when purchasing dairy products?

When buying dairy products, consumers take into account different sources of information, as shown in Figure 5. In most cases, this is the advice of relatives and friends and professional knowledge—27.1% and 26.4%, respectively. At the same time, there is no significant difference between these categories (*p* = .73). Differential analysis found that when buying dairy products, a statistically significant response (*p* < .001) has information from relatives/friends and their own professional knowledge compared to information from all other sources.

The same contribution to the decision to buy dairy products is made by advertising and internet sources—10.8% each (*p* = 1).

At the same time, when buying dairy products, there was no significant difference between the influence of advertising and school knowledge (*p* = .07), advertising and popular science publications (*p* = .05), Internet and school knowledge (*p* = .07), Internet and popular science publications (*p* = .05), school knowledge and popular science publications (*p* = .91), school knowledge and doctor's recommendations (*p* = .57), and school knowledge and TV shows (*p* = .21).

Only 2.7% of respondents do not include dairy products in their diet. When asked what could have influenced such a decision or the decision to limit the consumption of dairy products, 11.3% of the answers referred to professional knowledge. Also, when limiting the consumption of dairy products, 10.9% of consumers were guided by the doctor's recommendations; in 8.3% of cases, decisions were made on the advice of family and friends. In 6 cases of 100, the opinion of consumers was influenced by information from the Internet, and in 6 cases of 100, by popular science publications. To a lesser extent, the opinion of consumers on this issue was influenced by advertising, TV shows, and school knowledge, respectively, 1.5%, 1.1%, and 0.4% of the respondents answered this way.

A comparison of the answers to the questions “What kind of information do you consider when purchasing dairy products?” and “In the case you do not consume or limit your consumption of milk and dairy products, what kind of information influences your decision?” is presented in Table 1.

**TABLE 1:** Factors influencing the decision to consume dairy products.

Factors	Influence the decision (% of the total number of respondents)	
	Consume dairy products	Do not consume dairy products
Advice from family and friends	27.1 ± 0.02	8.3 ± 0.02
Professional knowledge	26.4 ± 0.04	11.3 ± 0.04
Advertising	10.8 ± 0.02	1.5 ± 0.01
Internet	10.8 ± 0.04	6.0 ± 0.03
School knowledge	7.0 ± 0.01	0.4 ± 0.01
Popular science publications	6.7 ± 0.03	6.0 ± 0.03
Doctor's recommendations	5.8 ± 0.02	10.9 ± 0.03
TV shows	4.3 ± 0.02	1.1 ± 0.02

Analyzing the data in Table 1 there was no statistical difference between the frequency of the influence of professional knowledge and doctor's recommendations (*p* = .87) and between professional knowledge and the advice of family/friends (*p* = .20) on the respondents' decision to limit the consumption of dairy products. Also in this matter, there are no significant differences between the doctor's recommendations and the advice of family and friends (*p* = .26), the advice of family/friends and Internet (*p* = .34), the advice of family/friends and popular science publications (*p* = .34), Internet and advertising (*p* = .06), popular science publications and advertising (*p* = .06), as well as advertising and TV shows (*p* = .87).

**FIGURE 6:**
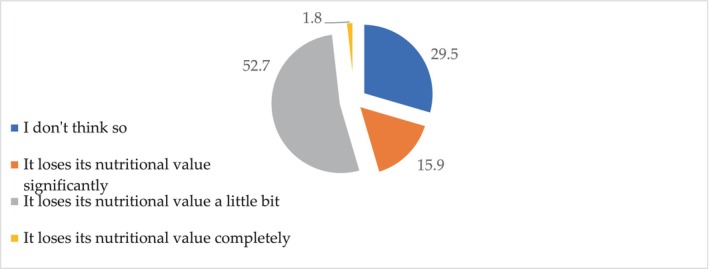
Distribution of responses (% of the total number of respondents) to the question: Do you agree that milk loses its nutritional value after being technologically processed at the factory?

In an attempt to find out what other reasons can influence the negative attitude toward milk and dairy products, the following question was asked: “Do you agree that milk loses its nutritional value after being technologically processed at the factory?” (Figure 6).

The majority of respondents (52.7%) believe that the nutritional value of milk after technological processing at the plant is somewhat reduced. Some respondents (15.9%) are sure that the nutritional value is significantly reduced and 1.8% believe that it is completely lost. Only 29.5% of respondents do not agree that milk loses its nutritional value after technological processing at the plant. Statistical analysis of all the answers to this question showed a high degree of their significance (*p* < .001).

Negative attitudes toward milk and dairy products may be caused by information about the falsification of milk and/or dairy products. Judging by the responses received, 61.4% (*p* < .001) of respondents met such information. However, as to the question of how many times a consumer has personally been faced with confirmed cases of falsified milk and dairy products, 73.2% of respondents answered “never”, 20.0% – “several” times, and 6.8% – “once”. All answers to this question corresponded to the specified level of significance (*p* < .001).

When specifying how the conclusion on the falsification of milk and/or dairy products was obtained, only 3.2% (*p* < .001) of the responses indicated: in laboratory studies or in an accredited laboratory.

When finding out whether consumers have an undesirable reaction from the gastrointestinal tract after taking milk and/or dairy products, more than half of the respondents (57.7%) did not report complaints, 36.4% of respondents sometimes face such a problem, and 5.9% often experience them. The variance analysis of the answers to this question confirmed the high statistical significance of all responses (*p* < .001).

Allergy to milk proteins was reported by 3.2% (*p* < .001) of respondents, but only 1.8% of respondents explained that it was proven by clinical methods. At the same time, the null hypothesis was not rejected in relation to the method of oral food intake and the test using serum containing immunoglobulin E. Both methods were chosen by 0.9% of respondents. Neither response specifies both methods together.

Similarly, lactose intolerance has a positive response in 3.6% (*p* < .001) of cases, while only 0.5% of survey participants (*p* < .001) have clinically proven confirmation of this fact.

Finding out the age at which one's lactose intolerance was detected did not have a statistically significant response. The frequency of responses to this question in the sample was distributed as follows: 0.9% of respondents chose “In the first months of life” response option, 0.9% of respondents chose “In preschool age” response option, 2.7% of respondents chose “In adolescence” response option, and 2.3% of respondents chose “After 30 years” response option.

To find out the satisfaction of consumers with the assortment of dairy products, a series of questions about consumers' taste preferences, composition, and nutritional value were asked.

Most consumers (49.1%) are completely satisfied with the assortment, and about the same 45.5% are satisfied to some extent. There was no statistical difference between these responses (*p* = .31). A significantly smaller part of the surveyed consumers 3.6% (*p* < .001) is not at all satisfied with the assortment of dairy products.

When asked about taste preferences, the majority of respondents (84.5%, *p* < .001) favored the traditional taste of dairy products. Only 15.5% (*p* < .001) of consumers are ready to purchase dairy products with new flavors. At the same time, in the answers to the question about added flavor and aromatic fillers in dairy products, preferences were divided almost equally: 53.2% of respondents are not satisfied with the presence of flavor additives, and 46.8% are satisfied. As a result, the significance level (p = 1.18) of the answers to this question does not reach the required value.

**FIGURE 7:**
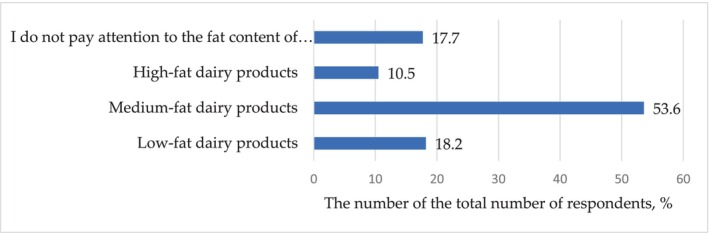
Distribution of responses (% of the total number of respondents) to the question: Do you pay attention to the fat content of dairy products?

Analyzing preferences in terms of products by nutritional value, it was found that 76.8% of consumers (*p* < .001) pay attention to the fat content of dairy products. At the same time, the majority of consumers (53.6%, *p* < .001) choose medium‐fat products (Figure 7). The answers have no statistically significant response: “Low‐fat dairy products” and “I do not pay attention to the fat content of dairy products” (*p* = .91), and “I do not pay attention to the fat content of dairy products” and “High‐fat dairy products” (*p* = .06).

At the same time, only 32.7% (*p* < .001) and 34.5% (*p* < .001) of respondents pay attention to the protein and carbohydrate content in dairy products, respectively, and 42.3% (*p* < .05) pay attention to the total caloric content.

**FIGURE 8:**
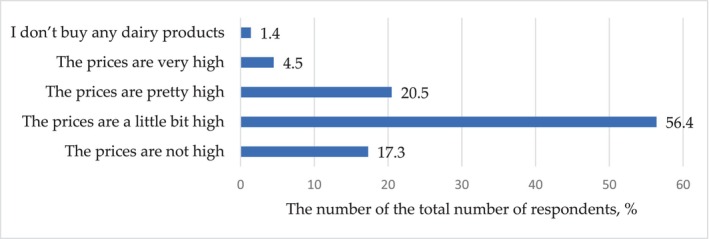
Distribution of responses (% of the total number of respondents) to the question: Do you think that the prices of dairy products are high in general?

Regarding economic considerations, 79.1% (*p* < .001) of respondents reported that the price of dairy products is important to them, and one‐fifth of consumers (20.5%, *p* < .001) consider this price to be quite high. Only 17.3% (*p* < .001) of respondents, as can be seen in Figure 8, believe that the price of dairy products is not at all inflated. At the same time, there was no statistically significant response difference between “quite high” and “not at all high” response options (*p* = .34) for the question on the price of dairy products.

**FIGURE 9:**
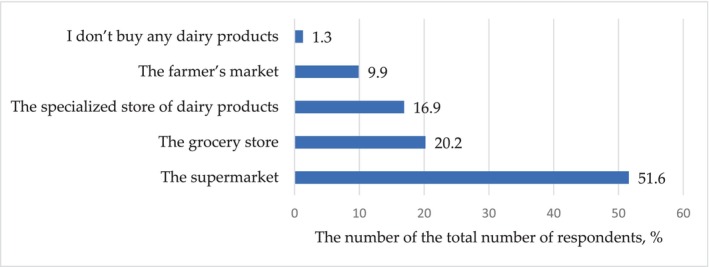
Distribution of responses (% of the total number of respondents) to the question: You buy milk and dairy products in.

The possibility of including dairy products in the diet can also be influenced by the place of their purchase. Figure 9 shows that a relatively small number of respondents (9.9%, *p* < .001) purchase dairy products on the market. In total, about 90% of respondents trust retail chains in this matter. More than half of consumers prefer grocery supermarkets (*p* < .001). Apparently, this is a general marketing pattern that allows, firstly, to purchase different products in one place and, secondly, to have a bigger choice in both manufacturers and assortment.

There is no significant difference between the answers to the question “You buy milk and dairy products” in grocery store or a specialized store of dairy products (*p* = .22).

Nevertheless, it is worth noting that 16.9% of those who took part in the survey prefer to buy dairy products in a specialized store of dairy products. This decision is consonant with the answer to another question: “Does the brand name of a dairy products manufacturer matter to you?” Out of all respondents, 76.4% (*p* < .001) pay attention to the brand name of a dairy products manufacturer.

## 4 DISCUSSION

The analysis of respondents by gender and age showed that the role of dairy products in nutrition is of greater interest to women than men. At the same time, it can be noted that older people, especially 60+, demonstrate a model of “fairly limited” consumption as opposed to a “filled” consumption model typical of young people and middle‐aged buyers. The insignificant percentage of participants over the age of 50 can be explained by the fact that, in general, the number of Internet users of this age in Russia is less than younger fellow citizens (Shpuntova, 2021).

The results of the study showed that the population understands the health and biological significance and nutritional value of milk and dairy products and regularly includes them in the diet. More than 50% of respondents consume dairy products daily. The choice of consumers is not limited to one type of product; milk, fermented milk products, cheese, cottage cheese, and butter are equally in demand. However, most of the population prefer traditional types of dairy products.

As for the preferences in the choice of products by nutritional value, it was found that first of all consumers pay attention to the fat content, and then to the total caloric content, protein, and carbohydrate content in dairy products. Apparently, these data indicate a lack of consumer awareness of the nutritional value and the role of macronutrients in human nutrition. Probably, only one‐third of consumers understands the relationship between and the role of proteins, lipids, and carbohydrates on human metabolism.

The fact that a significant number of consumers choose medium‐fat dairy products may indicate organoleptic preferences. Low‐fat dairy products are known to have a less pronounced taste, and in an attempt to combine the benefits and taste, consumers' choice falls in most cases on medium‐fat products.

The analysis of the reasons for the negative attitude toward milk and dairy products prompted the idea of insufficient awareness of customers about milk processing technologies and about the preservation of the nutritional value of dairy products.

If we take into account that the concept of “nutritional value” includes the content of proteins, fats, and carbohydrates, then most consumers are mistaken in the fact that milk and dairy products partially or completely lose their nutritional value after various technological operations.

Stereotypes about the shelf life of products and the presence of preservatives in them, replicated in the media, can repel some consumers from purchasing dairy products. Almost a quarter of respondents believe that the long shelf life of dairy products is always due to the introduction of preservatives into them, exactly the same number do not know the answer to this question. Consequently, almost half of consumers are not aware that modern technologies of processing and packaging of dairy products allow them to preserve products for a long time without deterioration of their quality indicators and without the addition of preservatives.

Negative attitudes toward milk and dairy products may be caused by information about the falsification of milk and/or dairy products. Indeed, such information sometimes appears in news reports, TV shows, or Internet sources, since the quality of milk and dairy products is strictly controlled by government agencies at various levels in Russia. Most respondents have never personally been faced with confirmed cases of falsified milk and dairy products. Comparing the answers related to the information on the falsification of milk and/or dairy products and the confirmation of cases of falsification of milk, it was concluded that in 95% of cases, negative information about milk and dairy products is unreliable.

One of the reasons for not consuming dairy products is the potential adverse health effects. However, studies have shown that the ideas about milk protein allergy and lactose intolerance are exaggerated compared to reality.

To confirm a clinical allergy to proteins, it is necessary to perform an analysis of the gold standard—oral food intake. However, in most places, it is difficult or practically impossible to perform it as a standard task, therefore, food allergy to milk proteins is either not confirmed or overdiagnosis is possible. In the case of lactose intolerance, fermented milk products and products with commercially hydrolyzed lactose are known to be indicated (Docena et al., 2016; Emura et al., 2020; Fleischer et al., 2011; Khoroshinina et al., 2017; Scheider et al., 2015).

The level of consumption of milk and dairy products by the population largely depends on influence marketing. When deciding to purchase dairy products or refuse to include them in their diet, consumers trust the advice from family and friends and professional knowledge much more than information from school knowledge, popular science publications, from a doctor's recommendations, and from TV shows.

In total, more than 20% of decisions on limiting the consumption of dairy products are made on the basis of information (advice from family/friends, Internet, popular science publications, advertising, TV programs, and school knowledge), which may be misunderstood by participants or not completely truthful.

One of the indicators of the economic and social development of the dairy industry is the purchasing power of household incomes. Most survey participants consider the prices of milk and dairy products to be a little bit high.

Processors note that a decrease in demand for dairy products leads to a decrease in processing volumes and, as a result, a decrease in the volume of purchases of raw materials from agricultural producers. At the same time, the growth of consumption of dairy products is largely obstructed by the pricing policy of retail chains, as a result, the price of raw milk is falling, but for the consumer it is growing.

The results of the study showed the increase in demand for milk and dairy products can also be influenced by the place of their purchase. About 90% of respondents make purchases in retail chains. More than half of consumers prefer supermarkets. The advantage of supermarkets is the possibility of round‐the‐clock access to products, which is especially convenient for busy people. In addition, supermarkets have products of different brands, which makes it possible to choose and compare prices. The presence of supermarket chains also allows for organizing promotions and offering discounts, which also attracts buyers.

Only a fifth of respondents prefer to buy dairy products in a specialized store of dairy producers. At the same time, most buyers pay attention to the brand name of a dairy products manufacturer. This indicates how important it is for the manufacturer to gain consumer trust and maintain the earned reputation.

In general, it can be argued that consumers do not have enough awareness about the benefits, nutritional value, and modern technologies for the production of dairy products. Perhaps more active promotion of popular science, educational programs, and educational courses about nutrition in general, and about dairy products in particular, will be an effective measure to improve the composition of the diets of the population.

## 5 CONCLUSIONS

The results of the survey allow us to identify the main reasons for the decline in milk and dairy product consumption and identify ways to solve them.

First, the decline in milk and dairy products demand and refusal are associated with information about milk and dairy products from not enough reliable sources.

Representatives of dairy industry in Russia are seriously concerned about the replication of negative information about milk and dairy products in the media and urge specialists in the field of medicine and milk processing to consolidate their efforts to educate consumers about the benefits of cow's milk. The Center for the Study of the Dairy Market (DIA) notes that any negative conversations on the topic of the quality of dairy products discourage the desire of the population to buy it (PRODUKT.BY, 2019).

According to representatives of the Research Agency GIRA, dairy industry specialists need to carefully create its advertising, agitate, and explain how important it is to consume dairy products. At the same time, it is necessary to use not only traditional sources of information, such as television and the press, but also new Internet sources, including social networks (Lafougere Christophe, 2019).

The purchasing power of the population should also be taken into account. For the majority of respondents, the price of dairy products is significant and it seems to be overpriced to the majority of respondents.

The expansion of the assortment and modification of the tastes of dairy products have a lesser impact on their consumption compared to the cost and information about the benefits.

According to the author, educational programs and increasing the purchasing power of the population can increase the demand for dairy products. Ultimately, this will have a positive impact on preserving the health of the nation.

## AUTHOR CONTRIBUTIONS

**Alla L'vovna Novokshanova:** Conceptualization (lead); data curation (lead); formal analysis (equal); funding acquisition (lead); investigation (lead); methodology (lead); project administration (lead); resources (equal); software (equal); supervision (equal); validation (equal); visualization (lead); writing – original draft (lead); writing – review and editing (equal). **Natalia Olegovna Matveeva:** Conceptualization (supporting); data curation (supporting); formal analysis (equal); funding acquisition (supporting); investigation (supporting); methodology (supporting); project administration (supporting); resources (equal); software (equal); supervision (equal); validation (equal); visualization (supporting); writing – original draft (supporting); writing – review and editing (equal). **Dmitry Borisovich Nikityuk:** Funding acquisition (supporting); investigation (supporting); project administration (supporting).

## FUNDING INFORMATION

The research was conducted with the financial support of the Ministry of Science and Higher Education of the Russian Federation (research project No. FGMF‐2022‐0002).

## CONFLICT OF INTEREST STATEMENT

The authors declare that they have no competing interests.

## Data Availability

The datasets generated during the current study are available from the corresponding author upon reasonable request by email.
